# Corticotomy in orthodontic treatment: systematic review

**DOI:** 10.1016/j.heliyon.2020.e04013

**Published:** 2020-05-27

**Authors:** Alina Apalimova, Àlvar Roselló, Enric Jané-Salas, Carlos Arranz-Obispo, Antonio Marí-Roig, José López-López

**Affiliations:** a Department of Odontostomatology, School of Dentistry, University of Barcelona, Barcelona, Spain; b Oral Surgery and Oral Implantology, School of Dentistry, University of Barcelona, Barcelona, Spain; c Medicine and Surgery, Oral Medicine at the School of Dentistry, Faculty of Medicine and Health Sciences, University of Barcelona, Barcelona, Spain; d Oral Health and Masticatory System Group (Bellvitge Biomedical Research Institute) IDIBELL, University of Barcelona, L'Hospitalet de Llobregat, Barcelona, Spain; e Department of Oral and Maxillofacial Surgery, Hospital Universitari de Bellvitge, Barcelona, Spain; f Surgical Medical Service, Barcelona University Dental Hospital, Barcelona, Spain

**Keywords:** Dentistry, Dental surgery, Dental materials, Periodontics, Oral medicine, Prosthetic dentistry, Corticotomy, CAOT, Orthodontic surgery

## Abstract

**Objective:**

The aim of this study is to evaluate corticotomies effects to accelerate or facilitate dental movements in different kind of orthodontic treatments.

**Data:**

This report followed the PRISMA Statement. A total of 9 articles were included in review.

**Sources:**

Two reviewers performed a literature search up to December 2018 in four databases: PubMed, Web of Science, Scopus and SciELO.

**Study selection:**

Controlled clinical trials and randomized controlled clinical trials conducted in human patients and published during the last 10 years in English were eligible to be selected. The articles should give detailed information about the results and treatment parameters. There were no limitations established in terms of the type of malocclusion to be corrected or the type of orthodontic treatment performed.

**Results:**

The methodological quality and evidence of the selected studies was low. Most of the studies observed a statistically significant increase in the rate of dental movement, when performing alveolar corticotomies as coadjuvants of orthodontic treatment; either with the conventional technique or with piezocision. The effect of combining corticotomy with bone grafts was assessed.

**Conclusions:**

High heterogeneity among studies made it difficult to draw clear conclusions. However, within the limitations of this review, the corticotomy procedures were able to statistically and clinically produce significant temporary decrease in orthodontic tooth movement rate. This technique does not seem to involve major complications compared to conventional orthodontic treatments.

**Clinical relevance:**

The use of this technique can reduce treatment time and therefore the undesirable effects associated with prolonged treatments.

## 1 Introduction

During the last decades the number of patients seeking orthodontic treatments has increased, as also have the aesthetic demands and the request for shorter treatment times [1].

Most conventional orthodontic treatments require almost two years to complete [2]. Several factors can influence the length of treatment, such as the severity of the case, the need of the extraction of premolars, the clinician's experience and of course patient's cooperation [3].

Different techniques, both surgical and non-surgical, are used as coadjuvants of orthodontic treatment [4]. Lately, one of the most used has been corticotomy. This is defined as a surgical procedure in which the cortical bone is cut, perforated or mechanically altered, without actually affecting the medullary bone [5]. The aim of this technique is to accelerate orthodontic dental movements in order to reduce treatment time and therefore to reduce the undesirable effects associated with prolonged treatments (root resorption, periodontitis, decalcification and gingival recession), in addition to increase patient's satisfaction [4, 6].

### 1.1 Biological bases

Bone remodeling processes begin when an orthodontic force is applied over the periodontium which, in turn, generates an aseptic inflammatory response [7]. This tissue response initially involves vascular changes, followed by the synthesis of prostaglandins, cytokines, growth factors, neurotransmitters, metabolites of arachidonic acid and hormones [7, 8]. The role of cytokines during tooth movement is not clear; however, it has been suggested that cytokines and other inflammatory markers, such as prostaglandin E2, may activate bone remodeling characterized by bone resorption in the compression region and bone deposition in the tension region of the periodontal ligament [9].

Orthodontic treatment duration depends on dental movement rate, which depends on alveolar remodeling rate. Therefore, it is considered possible to achieve an increase in the treatment speed, influencing the biological reactions of the alveolar bone, the periodontal ligament, the gingiva and the vascular and neuronal irrigation [10].

Corticotomy involves the creation of shallow perforations or cuts made in the cortical alveolar bone while the trabecular or medullary bone is left intact, in order to induce an acceleration of the normal physiological processes involved in bone healing [11].

Following the surgical bone wounding a "Regional Acceleration Phenomenon" (RAP) takes place [12]. RAP potentiates tissue reorganization and healing by a transient burst of localized hard and soft tissue remodeling [12], it is associated with a perfusion and bone turnover increase and a decrease in bone density [13].

It is similar to the processes associated with bone fractures healing, which includes a reactive phase, a reparative phase and a remodeling phase [14]. The reactive phase lasts 7 days and it is characterized by immediate constriction of blood vessels followed by hematoma within a few hours. The hematoma will form an aggregate of fibroblasts, intercellular materials and other supporting cells [7]. A few days later, the fibroblasts of the periosteum surrounding the lesion area and the fibroblasts from the granulation tissue will transform into chondroblasts and form hyaline cartilage [15]. Periosteal cells, peripheral to the injured area, will become osteoblasts and begin to form bone tissue. The association between hyaline cartilage and bone tissue is called "bone callus" and will be replaced by lamellar bone in a later phase [14, 15]. It is estimated that RAP reaches its maximum level in about 2–4 months. Coordination between the surgeon and the orthodontist is essential in order to achieve optimal results [16, 17].

Some studies have shown that low-level laser therapy, at a cellular level, causes an increase in RANKL in the periodontal ligament which, in turn, increases the differentiation of precursor cells into activated osteoclasts and potentially increases the rate of orthodontic tooth movement [11]. However, other studies show that low-energy laser irradiation does not accelerate tooth movement and can even slow it. The discrepancies may be explained by the different treatment protocols used in these studies, including the wavelengths of the lasers, irradiation doses, locations, and frequencies [18]. Some authors as for example Varella et al. [19] tried to identify and assess the gingival crevicular fluid levels of IL-1b during orthodontic tooth movement and the correlation with the use low-level laser therapy to determine whether it can accelerate orthodontic tooth movement, they observed that low-level laser therapy-facilitated orthodontics is approximately 2 times faster than conventional orthodontics and can be used as a non -surgical method to provide physical stimulation resulting in accelerated tooth movement. Gkantidis et al. [10] in the other hand concluded that there was weak evidence that low laser therapy plus a corticotomy were associated with accelerated orthodontic tooth movement. However, further research is required before the dual therapy achieves routine application.

### 1.2 Historical background

The concept of making cuts in the bone to facilitate tooth movement is not new. In 1959 Köle [20, 21] was the first to describe orthodontics facilitated by modern corticotomy. He based his technique on interproximal corticotomy cuts extended through the entire thickness of the cortical layer, barely penetrating into the medullary bone, since he considered that cortical bone was the one that provided the greatest resistance to dental movement [21]. These vertical cuts were connected beyond the apices of the teeth with a horizontal osteotomy cut, essentially creating blocks of bone in which one or more teeth were embedded. He believed that using the teeth crowns as handles he was able to move the bone blocks independently of each other as they were only connected by the less-dense medullary bone. This technique was known as “bony block” (tooth-bone unit) and caused almost four decades of confusion regarding the right dental movement mechanism [22, 23]. In 2001, Wilcko et al [12] suggested, based on computed tomographic studies, that the rapid tooth movement associated with corticotomy-facilitated orthodontics was more likely the result of a demineralization/remineralization process due to the cuts performed and not because of “bony block” movement.

The Wilcko brothers patented and trade marked their technique as Periodontally Accelerated Osteogenic Orthodontics (PAOO) [12]. PAOO consists of raising a mucoperiosteal flap, decorticating the vestibular and lingual/palatal areas of the alveolar bone and adding bone graft material under the periosteum [12]. It is necessary to place relatively high volumes of particulate bone-grafting material between the intact elevated periosteum and the opposing corticotomized bone [23]. This new volume of bone will facilitate a greater scope of tooth movements and reduce the need for extractions, while ensuring adequate periodontal support [24].

Within the different Corticotomy Assisted Orthodontic Techniques (CAOT) existing, Periodontally Accelerated Osteogenic Orthodontics (PAOO) was the first technique described in depth [25]. However, depending on each professional and specific case there might be numerous variations, either by the type of decortication pattern, if palatal cuts are made or not, or whether bone graft material is used.

### 1.3 Characteristics, applications and limitations

Among the main advantages of CAOT compared to traditional orthodontic treatments we may found an increase in tooth movement rate and limits, a decreased need for extractions, decreased treatment times, increased alveolar volume and a more structurally complete periodontum (correction of preexisting bony dehiscences and fenestrations) [26]. CAOT can be completed in a third or a quarter of the time required for traditional orthodontic treatment, reducing treatment time between 6 and 8 months [25, 27]. In addition, in combination with traditional orthodontics, segmental problems such as forced eruption of impacted teeth or molar intrusion, can be corrected in a shorter period of time [28]. CAOT techniques are also indicated in cases of space closure, open bite correction, cases of moderate or severe crowding, dento-alveolar bimaxillary protrusion treatments, class II malocclusion (requiring moderate expansion or extraction), and mild class III malocclusion [25, 29].

Although, corticotomy procedures are quite efficient and predictable, they are relatively invasive because of the requirement for full mucoperiostial flaps, bone injury and suturing, which potentially result in postsurgical discomfort and complications such as pain, swelling, slight interdental bone and attached gingiva loss or infection [28, 30].

Among CAOT's limitations we may find that ankylosed teeth cannot be reliably moved nor, can teeth be moved through devitalized bone, a situation that can occur in conjunction with long-term cortical steroid or bisphosphonate therapy [24].

Since its remarkable benefits and capabilities, specialists sometimes think of periodontally accelerated osteogenic orthodontics when nothing else works. Still, it must not be considered as a rescue technique but a tool to be used in certain patients according to their needs [24].

The aim of this study is to evaluate corticotomies effects, as a surgical coadjutant technique, to accelerate or facilitate dental movements in different kind of orthodontic tooth treatments.

## 2 Materials and methods

### 2.1 Search strategy and development of focused question

An electronic literature search up to December 2018 was performed in PubMed, Web of Science, Scopus and SciELO databases by two reviewers (AA and AR), the discrepancies were resolved by JLL and agreed upon with EJA, CAO and AMR. The PICO (Pacient, Intervention, Comparison and Outcome) question was: Does the orthodontic tooth treatment assisted by corticotomy decrease the total treatment time compared to conventional orthodontic tooth treatment? The reporting of these systematic review followed the PRISMA (Preferred Reporting Items for Systematic Review and Meta-Analyses) (http://www.prisma-statement.org/index.htm) guidelines for adequate conductance of systematic reviews.

### 2.2 Selection of studies

The following combination of keywords and Boolean operators were used: ("Surgical procedure" OR "Corticotomy") AND ("Tooth Movement Technique" OR "Orthodontic") AND (English [la]) NOT (letter [pt] OR comment [pt] OR editorial [pt]. In addition, a complementary manual search, of potentially relevant studies, was carried out through the references lists of the included and excluded articles.

### 2.3 Eligibility criteria

The articles included in this systematic review had to meet the following inclusion criteria: controlled clinical trials and randomized controlled clinical trials conducted in human patients and published during the last 10 years in English. The articles should give detailed information about the results and treatment parameters. There were no limitations established in terms of the type of malocclusion to be corrected or the type of orthodontic treatment performed. We excluded case reports, case series, systematic reviews, preclinical studies and clinical trials that provided insufficient information.

### 2.4 Quality assessment

The quality of the articles was assessed taking into account the Jadad scale [31], which evaluates 5 different points in relation to randomization, blinding and abandonment rate. The risk of bias was also assessed, using the Cochrane guidelines [32].

## 3 Results

### 3.1 Selection of studies

**Figure 1:**
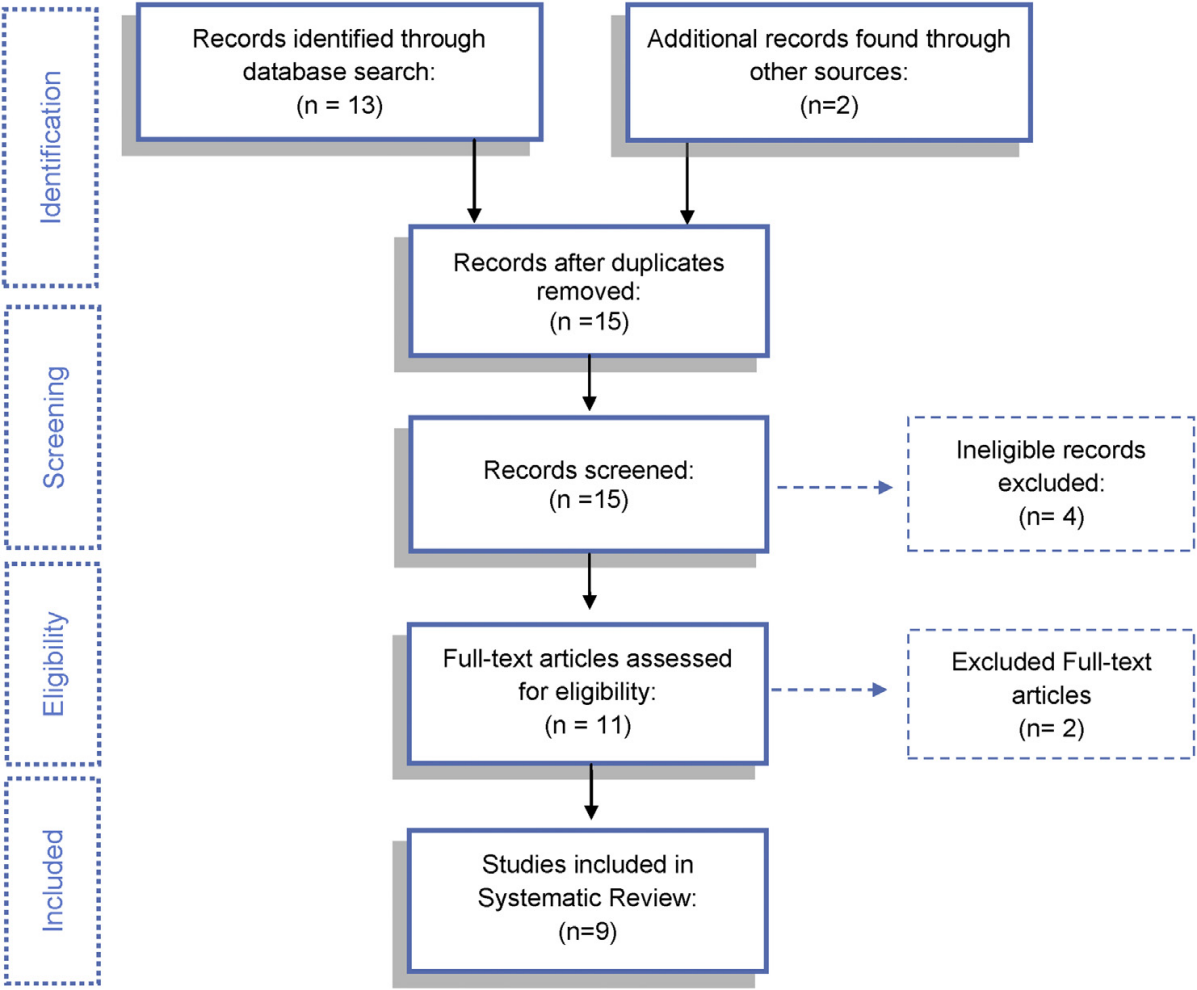
PRISMA flow chart.

The initial search was able to find 15 articles, 13 in MEDLINE database and 2 from a manual searching of reference lists, 11 of which were selected after a review of their titles and abstracts. Following a full text reading, 9 of the articles [33, 34, 35, 36, 37, 38, 39, 40, 41] met the inclusion criteria and were included in the review; 7 were randomized controlled clinical trials [33, 34, 35, 36, 37, 38, 39] and 2 were controlled clinical trials [40, 41] (Figure 1).

### 3.2 Quality of articles

All the analyzed articles would be considered low quality, since none of them was described as double blind and therefore did not exceed 3 points on the Jadad scale [31]. Those articles not described as randomized [40, 41] obtained the lowest score (Table 1). According to Cochrane [32] guideline, three studies would be considered to have low risk of bias [34, 35, 36]; on the other hand, the remaining six [33, 37, 38, 39, 40, 41] would have high risk of bias (Table 2).

**Table 1:** Methodological quality assessment.

	Was the study described as randomized?	Was the randomized scheme described and appropriate?	Was the study described as double-blind?	Was the method appropriate?	Was there a description of dropouts and withdrawals?	Quality
Abbas et al. [33], 2016	Yes	Yes	No	-	Yes	Low
Bahammam [34], 2016	Yes	Yes	No	-	Yes	Low
Charavet et al. [35], 2016	Yes	No	No	-	Yes	Low
Ma et al. [36], 2015	Yes	Yes	No	-	No	Low
Alikhani et al. [37], 2013	Yes	No	No	-	Yes	Low
Al-Naoum et al. [38], 2013	Yes	Yes	No	-	Yes	Low
Shoreibah et al. [39], 2012	Yes	No	No	-	Yes	Low
Shoreibah et al. [40], 2012	No	-	No	-	No	Low
Aboul-Ela et al. [41], 2011	No	-	No	-	Yes	Low

**Table 2:** Cochrane risk of bias assessment.

	Random sequence generation	Allocation concealment	Performance bias	Detection bias	Attrition bias	Reporting bias	Other sources of bias	Risk of bias
Abbas et al. 2016 [33]	**+**	**?**	**+**	**-**	**+**	**+**	**-**	**HIGH**
Bahammam 2016 [34]	**+**	**+**	**+**	**+**	**+**	**+**	**?**	**LOW**
Charavet et al. 2016 [35]	**+**	**?**	**+**	**+**	**+**	**+**	**-**	**LOW**
Ma et al. 2015 [36]	**+**	**?**	**+**	**+**	**+**	**+**	**?**	**LOW**
Alikhani et al. 2013 [37]	**+**	**?**	**-**	**+**	**+**	**+**	**?**	**HIGH**
Al-Naoum et al. 2013 [38]	**+**	**+**	**+**	**-**	**+**	**+**	**?**	**HIGH**
Shoreibah et al. 2012 [39]	**?**	**?**	**-**	**-**	**+**	**?**	**-**	**HIGH**
Shoreibah et al. 2012 [40]	**?**	**?**	**-**	**-**	**+**	**+**	**-**	**HIGH**
Aboul-Ela et al. 2011 [41]	**+**	**?**	**+**	**?**	**+**	**+**	**?**	**HIGH**

### 3.3 Study design

Standardized data extraction tables were created to summarize the data obtained from the articles. The characteristics of the study and participants, the techniques used, and the results obtained were assessed separately.

All the studies evaluated had a control group comparable to the study group; some articles performed Split Mouth techniques [33, 38, 41].

The selected studies used different methods to analyze variables such as bone density, tooth movement, root resorption or periodontal parameters (Table 3).

**Table 3:** Studies characteristics.

Author and Year/Study design	Nº Participants Study/Control	Withdrawals	Dental movement assessment	Periodontal parameters assessment	Bone density assessment	Root resorption assessment	Study duration
Abbas et al. 2016 [33]/RCT	S:10 C:10 + Split-Mouth	No	CBCT. Displacement and inclination of canines and molars	Plaque index, attachment level	--	CBCT	3 months
Bahammam 2016 [34]/RCT	S1: 11 S2:11 S3:11	4	--	Probing depth	XR	XR	3–5 months + 9 months follow up
Charavet et al. 2016 [35]/RCT	S:12 C:12	2	Digital measurement of interdental distance. Incisors inclination with cephalometry	Plaque index, probing depth, attachment level, bleeding, scars.	CT	CT	--
Ma et al. 2015 [36]/RCT	S:15 C:15	--	CT	--	CT	--	8 months
Alikhani et al. 2013 [37]/RCT	S:10 C:10	No	U3-U6 distance measurement in plaster models	Inflammatory response in crevicular liquid	--	XR	1 month
Al-Naoum et al. 2013 [38]/RCT	Split-Mouth 30	No	Digital measurement U3-U6 distance	--	--	--	3 months
Shoreibah et al. 2012 [39]/RCT	S:10 C:10	3	--	Probing depth	XR + DBSWIN software	XR	2–3 months + 6 months follow up
Shoreibah et al. 2012 [40]/CCT	S:10 C:10	--	--	Probing depth	XR + DBSWIN software	XR	Orthodontic treatment time + 6 months follow up
Aboul-Ela et al. 2011 [41]/CCT	Split-Mouth 13	2	Digital measurement U3-U6 distance	Plaque index, attachment level	--	--	4 months

S: Study Group. C: Control Group. RCT: Randomized Clinical Trial. CCT: Controlled Clinical Trial. CBCT: Cone Beam Computed Tomography. XR: Radiography. CT: Computed Tomography. U3: Upper canines. U6: Upper molars.

The most used technique to assess the dental movement rate was the measurement of the distance between canines and molars [33, 34, 37, 38, 41], the measurement was performed with digital caliper on plaster models [37, 38] or with a software program if digital models were used [35, 37, 38, 41]. Another study performed measurements using CBCT [33].

Different periodontal parameters were evaluated, the most repeated among studies were the probing depth [34, 35, 39, 40, 41] plaque index [33, 35, 41] and anchorage loss [33, 41]. In order to assess bone density, some studies chose to perform radiographs at different times of the investigation and afterwards evaluate them with the DBSWIN software (Vistascan System). In order to calculate it, each of the 256 values of the grey range were assigned to a level of bone density; a line midway between every two lower anterior teeth and parallel to the root surface was drawn, from the apex of the alveolar crest to the root apex. The grey value was recorded in three points of this line and the average of these 3 measurements was calculated to obtain the mean bone density value [39, 40]. Other studies assessed bone density through images obtained with TC [35, 36].

The degree of root resorption was assessed using different techniques such as CBCT [33], TC [35] or radiographs [35, 37, 39, 40]; measuring the distance between the cement-enamel junction and the root apex at different times of treatment.

### 3.4 Participants' characteristics

Among the 9 studies, a total of 210 patients, with an average age of 24.6 years (SD = 15–45) were included. The articles collected samples from 13 to 33 patients, all articles except one [41], collect samples equal to or greater than 20 participants (Table 4). The most common method of recruitment was through patients seeking orthodontic treatment in different Faculties of Dentistry.

**Table 4:** Participants' characteristics.

Author and year	Nº/gender/Mean Age	Recruitment method	Inclusion criteria	Exclusion Criteria
Abbas et al. 2016 [33]	M & W: 20 20	--	15–25 years. Need of extraction of 1.4 and 2.4 + canine retraction. Class II-I. No POT. No systemic disease affecting bone density. No evidence of bone loss. Probing depth <3mm. Attached gingiva: 1–2mm. Good oral health.	-
Bahammam 2016 [34]	M:10 W:23 21,2	Faculty of Dentistry, "King Abdulaziz University", Saudi Arabia.	18–27 years. Class I + moderate crowding (4–5mm). No POT. No systemic disease or pharmacologic treatment affecting bone density. No evidence of bone loss. Probing depth <3mm. Good oral health.	-
Charavet et al. 2016 [35]	M:9 W:15 30	Orthodontic Department, "Liège University Hospital", Belgium.	21–39 years. Need of orthodontic treatment (both arches). Minimal-moderate crowding. ASA I or II. No periodontal disease, periradicular condition or untreated caries. Good oral health.	Pharmacologic treatment. >10% loss of alveolar support. Gingival recession >2 mm. Smokers. Altered bone metabolism. Mental or motor disabilities. Pregnancy.
Ma et al. 2015 [36]	M:11 W:19 25,5	Oral Surgery Departament, "Shanghai Ninth People's Hospital", China.	High risk of injury to the inferior alveolar nerve. No systemic disease, pharmacologic treatment, periradicular condition, or tooth loss. No smoking.	-
Alikhani et al. 2013 [37]	M:8 W:12 25,8	Orthodontic Department, "New York University", USA.	18–45 years. Need of extraction of 1.4 and 2.4. Class II-I. No systemic disease, pharmacologic treatment or untreated caries. No evidence of bone loss. Probing depth <4mm. Gingival and plaque index ≤1. No smoking.	Poor hygiene. Evidence of bone loss. Extreme skeletal Class II, overjet ≥10 mm. Systemic disease. Long pharmacologic treatment. Past or current periodontal disease. Probing depth >4mm. Gingivitis or caries. Smoking habit.
Al-Naoum et al. 2013 [38]	M:15 W:15 20,04	Orthodontic Department, "University of Al-Baath Dental School ", Syria.	15–24 years. Class II-I and II-II. No POT. No systemic disease or upper jaw periodontal disease. Absence of craniofacial syndromes, cleft lip/palate or previous dentofacial traumas. Absence of canine restorative or endodontic treatment. Absence of structural or morphological canine abnormalities. Good oral health.	-
Shoreibah et al. 2012 [39]	M:4 W:16 24,5	Orthodontic Department, "Faculty of Dental Medicine for Girls, Al-Azhar University-Girls Branch", Egypt.	Class I Skeletal. Lower anterior teeth crowding (3–5mm) No POT. No periodontal disease. No pharmacological treatment. No previous periodontal surgeries. Adequate gingival thickness. Good oral health.	-
Shoreibah et al. 2012 [40]	M:3 W:17 22	Orthodontic Department, "Faculty of Dental Medicine for Girls, Al-Azhar University-Girls Branch", Egypt	Class I Skeletal. Lower anterior teeth crowding (3–5mm) No POT. No periodontal disease. No pharmacologic treatment. No previous periodontal surgeries. Adequate gingival thickness. Good oral health.	-
Aboul-Ela et al. 2011 [41]	M:5 W:8 19	-	Need of extraction of 1.4 and 2. 4 + Canine retraction. Class II-I. No POT. No systemic disease affecting bone density. No evidence of bone loss. Probing depth<3mm. No attachment loss. Good oral health.	-

M: Men. W: Women. POT: Previous Orthodontic Treatment.

### 3.5 Inclusion criteria of the studies

The most common inclusion criteria among the studies were: absence of previous orthodontic treatment of any kind [33, 34, 38, 39, 40, 41]. Presence of class II malocclusion, division I or II [33, 37, 38, 41] or crowding [34, 35, 39, 40]. The absence of systemic diseases or regular administration of medication that could affect bone metabolism [33, 34, 36, 37, 38, 40]. Absence of radiographic evidence of bone loss [34, 37, 41]. Absence of periodontal disease or probing depth >3mm [33, 34, 35, 36, 37, 38, 39, 41]. Presence of good oral hygiene [33, 34, 35, 38, 39, 41]. Absence of active caries or endodontic treatment in the teeth to be moved [33, 37, 38].

### 3.6 Exclusion criteria of the studies

The most common exclusion criteria were: presence of any systemic disease or medical treatment that affect bone metabolism [37]. Presence of periodontal disease or loss of anchorage [37]. Poor oral hygiene [35, 37]. Evidence of alteration in bone structure or density [37], smoking habit [37], pregnancy [35], active caries [37] and mental or motor disability [35].

### 3.7 Studies results (Table 5)

#### 3.7.1 Concept of study

In the selected studies the effectiveness of conventional orthodontic treatment was compared with orthodontics assisted by corticotomy or piezocisión [33, 35, 36, 37, 38, 39, 40, 41]. The effect of bone grafts was also evaluated [34, 39]. Variables such as tooth movement, treatment time, bone density and root resorption were studied as well (Table 5).

**Table 5:** Characteristics and results of the studies.

Study	Objective	Maloclusion	Intervention	Orthodontic tt	Duration tt	Rresults
Abbas et al. 2016 [33]	Ortho vs Ortho + Corticotomy vs Ortho + Piezocision	Class II- I	**Corticotomy:** *Flap****:*** V 4mm from GM, from M U2 to M U4. *Cuts:* Vertical M and D of U3 + MOPs **Piezocision**: Cuts M and D to U3, 2mm from GM.	**Alignment**: Brackets 022″, finishing with SS archwire 16 × 22″ **Canine retraction**: NiTi spring + 150gf. **Force application:** Immediately post-corticotomy **Orthodontic control**: Every 2 weeks		Canine retraction in 3 months: Ortho (0,60mm), Corticotomy (1,22mm), Piezocision (0,99mm). Higher root resorption in C than S group.
Bahammam 2016 [34]	Corticotomy vs Corticotomy + bovine xenograft vs Corticotomy + bioactive glass	Class I + Moderate crowding	**Corticotomy:** *Flap:* V distal to L3. *Cuts:* With SS round bur. Verticals, 1–2 mm from alveolar crest and teeth apex. **Bone grafts**: S1 (no graft) S2(Bovine xenograft), S3 (Bioactive glass) Antibiotic tt + Analgesic tt + Diuretic tt for 7 days	**Alignment**: Brackets 022” + NiTi archwires 0,012″, 0.014″, 0.016″ y 0.018″, finishing with SS archwire 16 × 22″ **Force application:** 2 weeks post-cort. **Orthodontic control:** Every 2 weeks **Periodontal control**: Every month	S1: 15 weeks S2: 16,8 weeks S3: 14,1 weeks	Bone Density pre-Tt vs post-Tt S1: **-** 29.82%, S2: **-** 14.43%, S3: **-** 24.04%. Bone Density post-Tt vs 9 months post-Tt. S1: **+** 0.87%, S2: **+** 31.99%, S3: **+**13.71%. Bovine xenograft better than bioactive glass.
Charavet et al. 2016 [35]	Ortho vs Ortho + Piezocision	Light crowding	**Piezocision:** Vertical interradicular cuts, 5mm long and 3mm deep. Analgesic tt + CHX rinses 0.2% 7 days	**Alignment**: Damon System, with NiTi archwires 0,014″, 0.018″, 0.014” × 0.025″, 0,018”×0,025”, finishing with SS archwire 19 × 25”. **Force application**: 1 week pre- corticotomy **Orthodontic control:** Every 2 weeks		Treatment time 43% longer in ortho than ortho + corticotomy. No changes in recessions, root resorption, bone density or fenestrations.
Ma et al. 2015 [36]	Ortho vs Ortho + Corticotomy	Impacted mandibular third molars	Mucoperiosteal flap and occlusovestibular osteotomy. **Piezocision**: Vertical interradicular cuts, 2mm from alveolar crest + horizontal apical cut. Antibiotic tt + Analgesic tt. CHX rinses 0.12%.	**Distalization***(mesially or horizontally impacted molars*): Hook boned to impacted tooth and attached to a 0.016″ SS spring welded to a band in L7. **Extrusion**: Cantilever 17 × 25 ″of the main arch. **Orthodontic control:** Every month with CT.	S: 4 months C:7,5 months	Mean Treatment Time: C: 7, 5 months S: 4 months. No difference in surgery time, extraction time or complications.
Alikhani et al. 2013 [37]	Ortho vs Ortho + Piezocision	Class II-I	U4 extraction 6 months before. **Piezocision**: Three MOPs D to 1.3 or 2.3 (random)	**Alignment**: Brackets 022″, finishing with SS archwire16 × 22”. **Canine retraction**: NiTi spring + 100gf + traction from microimplant (between U5-U6)		Canine retraction 2.3 times greater in S than C and contralateral side.
Al-Naoum et al. 2013 [38]	Ortho vs Ortho + Corticotomy	Class II-I y II-II	U4 extraction 4 weeks before. **Corticotomy**: *Flap*: V and P. *Cuts*: vertical, interradicular, 1–2 mm from alveolar crest + horizontal cut 2–3 mm suprapical on V and palatal sulcus level on P + MOPs V and P	**Alignment**: Brackets 022″, finishing with SS archwire19 × 25”. **Canine retraction**: NiTi spring + 120gf. **Force application:** Immediately post-corticotomy **Orthodontic control:** Every 2 weeks		Dental movement time: 2–4 faster in S than C the first week post-corticotomy. No differences between genders.
Shoreibah et al. 2012 [39]	Corticotomy vs Corticotomy + bone graft	Class I + Moderate crowding.	**Corticotomy***:* *Flap:* V distal to L3. *Cuts*: With SS bur, vertical, interradicular,1–2 mm from alveolar crest and teeth apex. **Study group: +bone graft** Antibiotic tt + Analgesic tt + Antidematous tt for 7 days. CHX rinses 0.12% for 14 days.	**Alignment**: Brackets 022″, finishing with SS archwire 19 × 25”. **Force application:** Immediately post-corticotomy **Orthodontic control:** Every 2 weeks	S: 16,67 weeks C: 17 weeks	Average bone density increase: S: 128,3% C: 47,6% 6 months post- Tt vs pre-Tt. S: **+**25, 85%. C: **-**17,59%
Shoreibah et al. 2012 [40]	Ortho vs Ortho + Corticotomy	Class I + Moderate crowding.	**Corticotomy***:* *Flap:* V distal to L3. *Cuts*: With SS bur, vertical, interradicular, 1–2 mm from AC and teeth apex. Antibiotic tt + Analgesic tt + Antidematous tt for 7 days. CHX rinses 0.12% for 14 days.	**Alignment**: Brackets 022″, finishing with SS archwire 19 × 25”. **Force application:** Immediately post-corticotomy **Orthodontic control:** Every 2 weeks	S:17,5 weeks C:49 weeks	Bone density 6 months post- Tt: S: **-** 21,8% C:**-** 37,2%. CT: S: 1.5 ± 0.9 mm. C: 1.7 ± 9.5 mm
Aboul-Ela et al. 2011 [41]	Ortho vs Ortho + Corticotomy	Class II-I + Augmented overjet.	Extraction of 1st U4 a day before + 2nd U4 corticotomy day. **Corticotomy:** *Flap:* V 4mm from GM, from M U2 to M U4 *Cuts*: With SS bur, MOPs from U2 to U5	**Alignment**: Brackets 022″, finishing with SS archwire16 × 22”. **Canine retraction**: NiTi spring + 150gf + traction from microimplant (between U5- U6)		Dental movement rate 2 times higher in ortho + corticotomy. during the first 2 months. 1.6 times greater the 3rd month and 1.06 times greater the 4th month.

S: Study Group. C: Control Group. V: Vestibular. M: Mesial. D: Distal. U: Upper. L: Lower. GM: Gingival Margin. MOPs: Microperforations. Tt: Treatment. gf: Gram-force. Ortho: Orthodontic treatment. SS: Stainless Steel. CT: Computed Tomography. tt: treatment.

#### 3.7.2 Applications

The studies treated different types of malocclusion such as Class II division I [32, 37, 38, 41], Class II division II [38], Class I skeletal [39, 40] with light or moderate crowding [34, 35, 39, 40] or mandibular third molars impaction [36]. Several treatments plan included exodontia of premolars, in some cases these were performed 4–6 months before the surgical intervention [37, 38], other studies performed the extraction of one of the premolars the day before the intervention and the second one at the same moment of the corticotomy [41].

#### 3.7.3 Surgical procedure

Intracrevicular vestibular flaps [34, 36, 39, 40] or submarginal flaps [32, 41] were performed depending on the study; only one study performed both flaps, vestibular and palatal [38]. In piezoelectric corticotomy, no flaps are needed; a cut is made with a piezoelectric tip through the attached gum, 2 mm from the gingival margin to preserve the papilla. The bone cuts are made parallel to the roots of the teeth that need to be moved [35, 37].

In all cases, corticotomies were performed as a one-stage procedure, under local or trunk anesthesia [36], the cuts carried out were vertical, interradicular, 1–2 mm from the alveolar crest and the roots apex. In some cases, an apical horizontal cut [36, 38] or microosteoperforations (MOPs) [33, 37, 38, 41] were performed.

#### 3.7.4 Orthodontic treatment

In several studies, orthodontic leveling and alignment were carried out prior to corticotomy [33, 37, 38, 41]. Bahammam [34], chose to apply the orthodontic force two weeks after the surgical intervention; in other cases, orthodontic traction and corticotomy were executed together from the beginning [34, 35, 36, 39]. In general, the orthodontic treatment was carried out with Brackets 0.22 ″, using a sequence of NiTi arches and finished with a steel arch, either 16 × 22" [33, 34, 37, 41] or 19 × 25" [35, 38, 39, 40]. Canine retraction was carried out with a NiTi spring [33, 37, 38, 41], in two cases with the help of microimplants placed between the second premolars and the first upper molars [37, 38].

#### 3.7.5 Duration of studies

The duration of the studies was very variable since it depended on the time of orthodontic treatment, and therefore on each case complexity. In a few cases, a 6–9 months follow-up was carried out once the orthodontic treatment was finished [34, 39, 40].

## 4 Discussion

Most of the included studies in this systematic review were considered to have low quality evidence and a high risk of bias, besides having small samples. There was a high level of heterogeneity regarding studies design, clinical indications, treatment plans, surgical techniques used, orthodontic treatment method, orthodontic forces applied and duration of the studies. Due to this lack of homogeneity, which complicated the analysis and summary of the results, it was not possible to assess the data quantitatively as a meta-analysis.

In the recent years numerous review articles, case reports and case studies have been presented, but these texts do little to summarize the clinical implications of this procedure, others simply expose the different results without comparing them [42, 43, 44] or study corticotomy and piezocision techniques separately [45].

There is a lack of consensus in the literature about the description of a standardized CAOT technique. Wilcko et al. [13] were the first to thoroughly describe their method named PAOO, but currently there are many variations of CAOT, either by the type of flap performed, by the corticotomy pattern, the instrument used to make the cuts or by the use or not of bone grafts.

All randomized clinical trials included in this review used the oral sides as preferred areas of intervention, except for Al-Naoum et al. [38] who performed both oral and palatal corticotomies in 30 patients. All cuts were made through the cortical bone without fracturing or damaging the medullary bone underneath.

Several studies [35, 36, 38, 40] observed an acceleration of orthodontic dental movement statistically significant in short term. However, as studies were carried out on different types of malocclusion, ranging from light anteroinferior crowding [35, 40], to class II division II [38] or extrusion of impacted mandibular third molars [36], the average treatment time between studies cannot be compared.

For example, Ma et al. [36] when applying only orthodontic treatment in 15 patients obtained an extrusion average time of 7.5 months; the average time decreased to 4 months in those 15 patients receiving orthodontic treatment combined with corticotomy. On the other hand, Shoreibah et al. [40] obtained a longer treatment time (4.5 months) treating cases with anteroinferior crowding in 20 patients with class I skeletal.

Different types of periodontal approach can be used, either by raising a mucoperiosteal flap [33, 34, 36, 38, 39, 40, 41] or by making the cuts or MOPs directly through the periodontal tissue, for example in case of using piezocision [35, 37], another option is vestibular incision subperiosteal tunnel access (VISTA) [46], which consists of vestibular incisions followed by elevation of the full-thickness subperiosteal tunnel flap. The studies that performed corticotomy with flap elevation opted for intracrevicular flaps [34, 36, 39, 40] or submarginal flaps [33, 41] to preserve the papilla. The cuts were vertical, interradicular, 1–2 mm from the alveolar crest and the root apex. In some cases, an apical horizontal cut [38] or microperforations (MOPs) [33, 37, 38, 41] were carried out^.^ In order to perform such cuts, the use of handpiece burs or piezoelectric tips is reported, but no specific indications were found for one instrument or another.

Both techniques present improvements in treatment time compared to conventional orthodontic approach. Charavet et al. [35] reported that the control group (n = 12), which did not undergo corticotomy by piezocisión, needed 43% more time to complete the treatment than the study group (n = 12). Moreover, Alikhani et al. [37] determined that canine retraction was 2.3 times greater in those 10 subjects who underwent microperforations by piezocision, compared to the control group (n = 10) to which conventional orthodontics were applied. Abbas et al. [33] contrasted the results obtained by conventional corticotomy and piezocisión corticotomy, they observed that the conventional corticotomy group (n = 10) exhibited higher rates of coronal movement during the first three months compared to the piezocision group (n = 10). These differences could be explained by the more extensive surgery required for the corticotomy procedure, which could have increased the intensity of RAP. This assumption is consistent with Wilcko et al. [47] who demonstrated that bone injury severity is directly related to the velocity of dental movement.

In a similar way, Al-Naoum et al. [38] in their split-mouth study of 30 patients reported that dental movement rate was four times higher on the study side during the first three days and three times higher from day five; they did not report differences between genders. This variation in the movement rate is consistent with other authors results, such as Aboul-Ela et al. [41] who, in another split-mouth study (n = 13), observed a dental movement rate two times greater during the first two months on the side in which the corticotomy was performed. This value, however, decreased to 1.6 from the third month and to 1.06 from the fourth month after intervention. In addition, Charavet et al. [35] observed that the time required to change each orthodontic arch was fewer at the beginning than in later stages. All this could be explained by the intensity decrease of RAP effect during the later months after the surgical procedure.

The maxillary expansion might compromise buccal bone thickness and produce root dehiscence or fenestration. It has been stated that this problem might be avoided if bone grafts are placed during corticotomy. Some studies evaluated bone density before and after corticotomy procedures, although their evaluation methods varied significantly [34, 35, 36, 39, 40]. Only two of the studies [34, 39] included in this review compared changes in bone density after corticotomy with and without the use of bone grafts. Shoreibah et al. [39] observed that bone density decreased by around 41.5% in both groups during the active dental movement period. Six months after the end of the treatment, control group's (n = 10) bone density was 17% lower than at the beginning, in the corticotomy group (n = 10), instead, there was an increase of 26%. Bahamman [34] studied two different types of grafts, bovine-derived xenograft and bioactive glass. He observed a decrease between 14%-30% in bone density rate during active dental movement period, in all 3 groups of 11 participants. The results obtained at the end of the treatment, and those obtained 9 months after were contrasted; 0.87% increase in bone density was observed in the control group, 32% in the bovine graft group and 14% in the bioactive glass group. Contrasting the initial values with those obtained 9 months after the end of the treatment, the bovine graft's group was the one obtaining the highest total increase in bone density.

In 2010, Teixeira et al. [8] observed in a study conducted in rats, that cortical bone perforations increased the expression of 37 different inflammatory cytokines. This discovery is important since, in humans as in rats, cytokines play an important role recruiting osteoclasts and therefore in the activation of bone remodeling machinery. In the same way, Alikhani et al. [37] studied different cytokines and chemokines variations in the crevicular fluid of 20 patients under orthodontic treatment, in different treatment stages, and contrasting concentrations obtained in these areas to contralateral zones not subjected to orthodontic forces. They also performed microperforations by piezocisión, 24 h later there was an increase of cytokines and chemokines concentration in both groups, although the group receiving microperforations had a greater inflammatory expression increase. After 7 days, cytokines and chemokines levels were still higher in the study group; the values were higher than those obtained at the beginning of the study, but lower than those collected after 24 h. The measurements carried out four weeks after the treatment startet showed that, although the values were higher in the study group, the difference between the two groups were not statistically significant; the inflammatory proteins concentrations had returned practically to the initial values.

In addition to the existing evidence that corticotomy improves and accelerates dental movement, the present systematic review found that CAOT is a safe procedure without significant effects on probing depth, nor attachment level [35, 41], bone density [34, 37, 40], or root resorption [33, 34, 35, 40]. Bahammam et al. [34] were the only ones who evaluated the relapse rate, after 9 months, only 27 of the 33 patients were available for clinical and radiographic reevaluation; these patients had a good clinical outcome and did not experience any regression. In 1959, Köle [20, 21] did not observe root resorption in any case, but erroneously attributed it to the fact that there was no tooth movement but a “bony block” one. Nowadays, the resorption rate is considered to be lower due to the demineralized state of bone during treatment time and the fact that normal and controlled orthodontic forces are applied. Therefore, a rapid movement occurs due to the lack of bone resistance and not because of excessive orthodontic force [17, 24].

Abbas et al. [33] and Shoreibah et al. [40] observed a lower degree of root resorption in the study group than in the control group. However, the two-dimensional limitations of periapical radiographs make these results unreliable.

Only three studies examined the degree of discomfort associated with corticotomy procedures. Charavet et al. [35] found that apprehension levels before treatment were the same in all 12 participants of each group. Once finished, the participants who underwent the piezocision were more satisfied and would undergo the same treatment again. Alikhani et al. [37] observed that the 10 participants from both groups reported discomfort during the first 7 days, but there were no statistically significant differences between them. Finally, Al-Naoum et al. [38] reported that 50% of participants had extreme pain when eating and 80% had severe inflammation the day after surgery on the corticotomy side. However, after 7 days there were no reports of extreme pain or inflammation; although this technique caused some discomfort, patients considered that the procedure was less traumatic than dental extraction.

In this review we have focused on surgical techniques such as corticotomy, but there are multiple other non-surgical techniques such as low-level laser therapy or photobiomodulation that can also accelerate tooth movement rate, minimize the treatment time and reduce comorbidities such as pain. Eslamial et al. [48] studied the effect of 810-nm continuous wave low-level laser therapy (LLLT) on the pain stemmed from orthodontic elastomeric separators and demonstrated that LLLT was effective in reducing the postseparation pain during the first 3 days, but not thereafter.

Fernandes et al. [49] studied the effect of photobiomodulation (PBM) on tooth movement rate and observed that PBM accelerates tooth movement during molar intrusion, by modulating the levels of cytokines (IL-1β, IL-6 and IL-8) and the total treatment time was significantly reduced in the irradiated group, compared with the non-irradiated.

## 5 Conclusions

Corticotomy procedures performed even with conventional methods or piezocision involve a rate increase in dental movement and acceleration during the first months, subsequently returning to baseline values.

Traditional corticotomy technique, performed with surgical burs and handpieces, obtain faster results than piezocision. However, it is more invasive and can lead to more surgical morbidity.

The CAOT procedures do not seem to involve major complications, such as root resorption, affection at periodontal level or pulpal vitality, in comparison to conventional orthodontic treat ments.

Bone density may increase as a result of simultaneous placement of bone grafting materials during corticotomy procedure.

Nowadays, the available literature about orthodontics facilitated by corticotomy techniques provides low quality evidence, which is why more research is needed. A research with less risk of bias would allow greater comparisons and more significant conclusions.

## Declarations

### Author contribution statement

All authors listed have significantly contributed to the development and the writing of this article.

### Funding statement

This research did not receive any specific grant from funding agencies in the public, commercial, or not-for-profit sectors.

### Competing interest statement

The authors declare no conflict of interest.

### Additional information

No additional information is available for this paper.
